# Supplementary material for: Dengue virus-reactive CD8+ T cells mediate cross-protection against subsequent Zika virus challenge
Source: Nat Commun. 2017 Nov 13;8:1459. doi: 10.1038/s41467-017-01669-z (PMC5682281; doi:10.1038/s41467-017-01669-z)
Supplement: Supplementary file 1 — Supplementary Information [file 41467_2017_1669_MOESM1_ESM.pdf]

Dengue Virus-Reactive CD8+ T Cells Mediate Cross-Protection Against Subsequent Zika Virus challenge

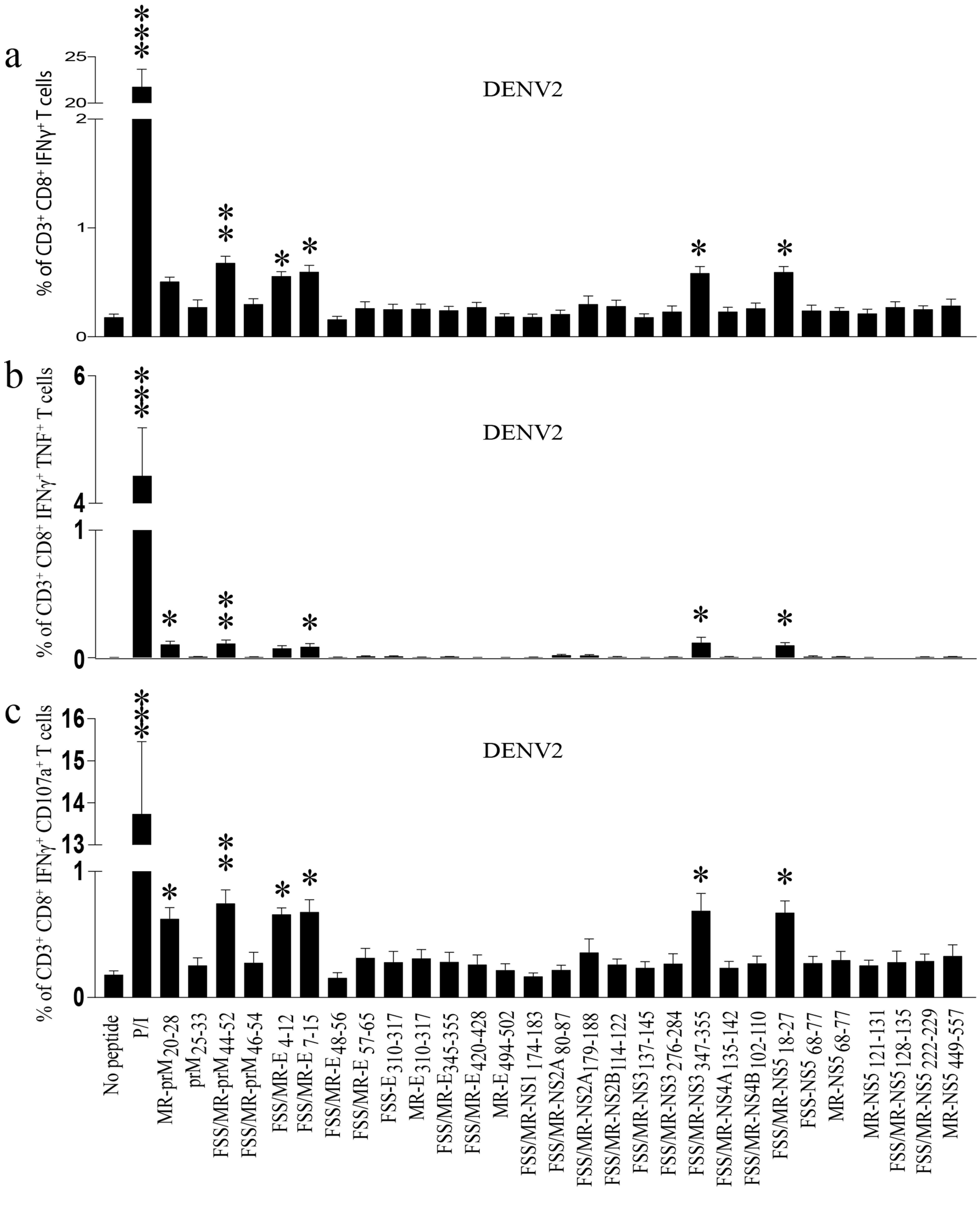

Supplementary Figure 1 Identification of DENV2-cross-reactive ZIKV epitopes recognized by $CD8^+$ T cells in *Ifnar1$^{-/-}$* H-2^b mice

Ifnar1$^{-/-}$ mice were infected retro-orbitally with DENV2 (2×10^4 FFU) (n=6) for 7 days. Splenocytes were stimulated with each of 29 previously identified ZIKV $CD8^+$ T-cell epitopes [1] and the frequencies of peptide-specific $CD3^+$ $CD8^+IFN\gamma^+$ T cells (a), $CD3^+CD8^+IFN\gamma^+TNF^+$ T cells (b), and $CD3^+CD8^+$ $IFN\gamma^+CD107a^+$ T cells (c) were detected using ICS assay. Data were pooled from two independent experiments with n = 3 mice per group per experiment and expressed as mean ± SEM. *p<0.05, **p<0.01, ***p<0.001. A Kruskal-Wallis one-way ANOVA was performed. P/I denotes PMA/ionomycin; FSS denotes ZIKV strain FSS13025; MR denotes ZIKV strain MR766. Supplementary Tables 1 and 2 provide exact values of *n* and *P*.

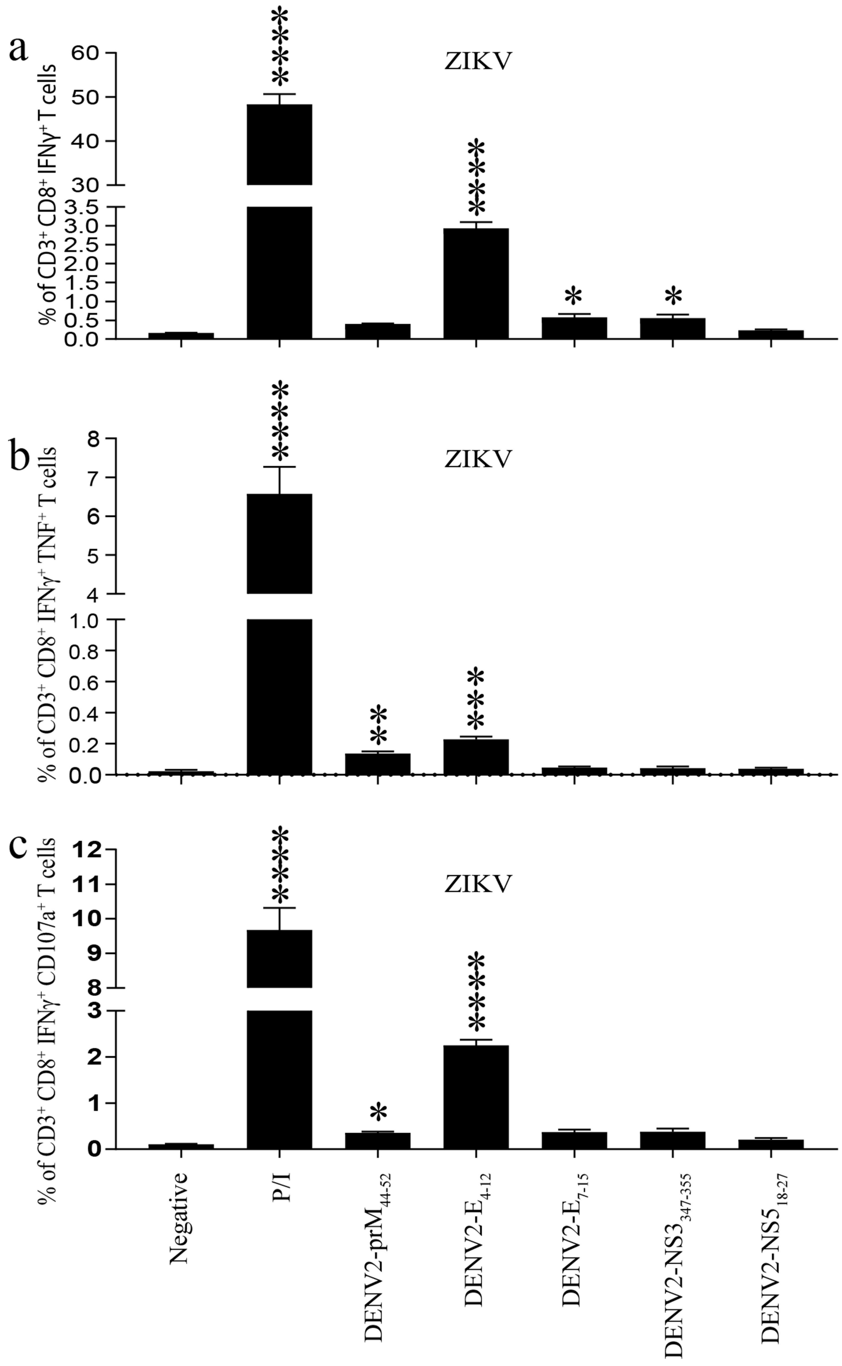

Supplementary Figure 2 Identification of ZIKV-cross-reactive DENV2 epitopes recognized by $CD8^+$ T cells in *Ifnar1*$^{-/-}$ H-2^b mice

Ifnar1$^{-/-}$ mice were infected retro-orbitally with ZIKV (1×10^2 FFU) (n=7) for 7 days. Splenocytes were stimulated with each of 5 DENV2 variants of DENV2-cross-reactive ZIKV $CD8^+$ T-cell epitopes. The frequencies of peptide-specific $CD3^+CD8^+IFN\gamma^+$ T cells (a), $CD3^+$ $CD8^+IFN\gamma^+TNF^+$ T cells (b), and $CD3^+$ $CD8^+IFN\gamma^+CD107a^+$T cells (c) were detected using ICS assay. Data were pooled from two independent experiments with n = 3-4 mice per group per experiment and expressed as mean ± SEM. *$p<0.05$, **$p<0.01$, ***$p<0.001$, ****$p<0.0001$. A Kruskal-Wallis one-way ANOVA was performed. Supplementary Tables 1 and 2 provide exact values of *n* and *P*.

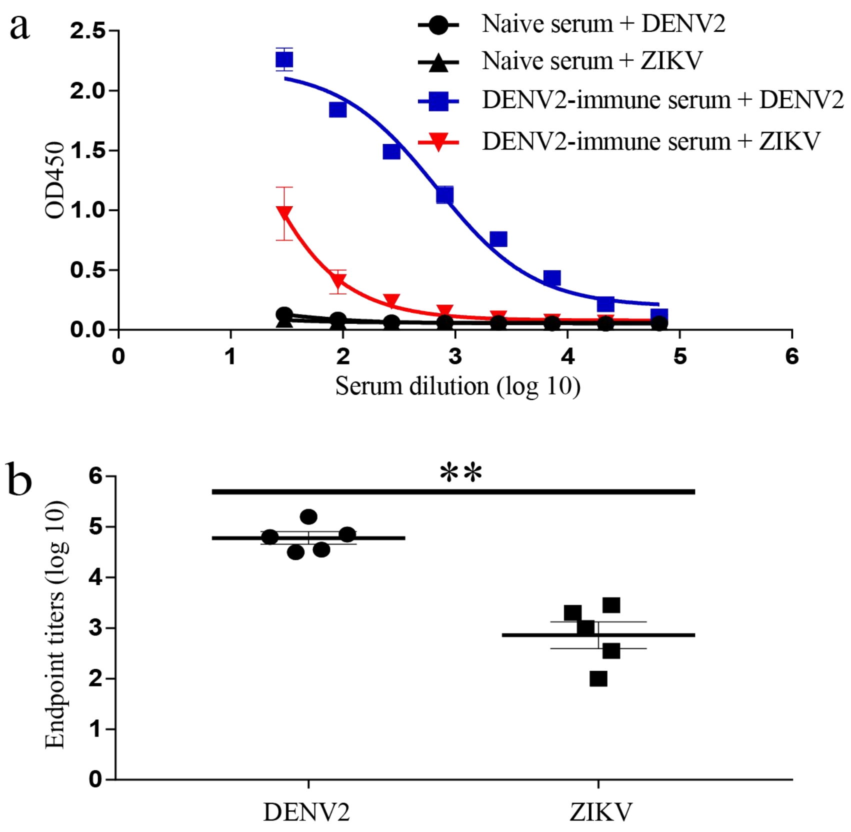

Supplementary Figure 3 DENV2-immune sera cross-reactivity with ZIKV

The binding capacity of sera from 5 naïve and 5 DENV2-immune *Ifnar1*$^{-/-}$ mice with DENV2 strain S221 and ZIKV strain FSS13025 was measured using capture ELISA (**a**). Reciprocal dilution endpoint titers of DENV2-immune serum against DENV2 and ZIKV were determined from the capture ELISA results (**b**). Data are expressed as mean ± SEM. $^{**}p<0.01$. Two-tailed Mann-Whitney test was performed. Supplementary Tables 1 and 2 provide exact values of *n* and *P*.

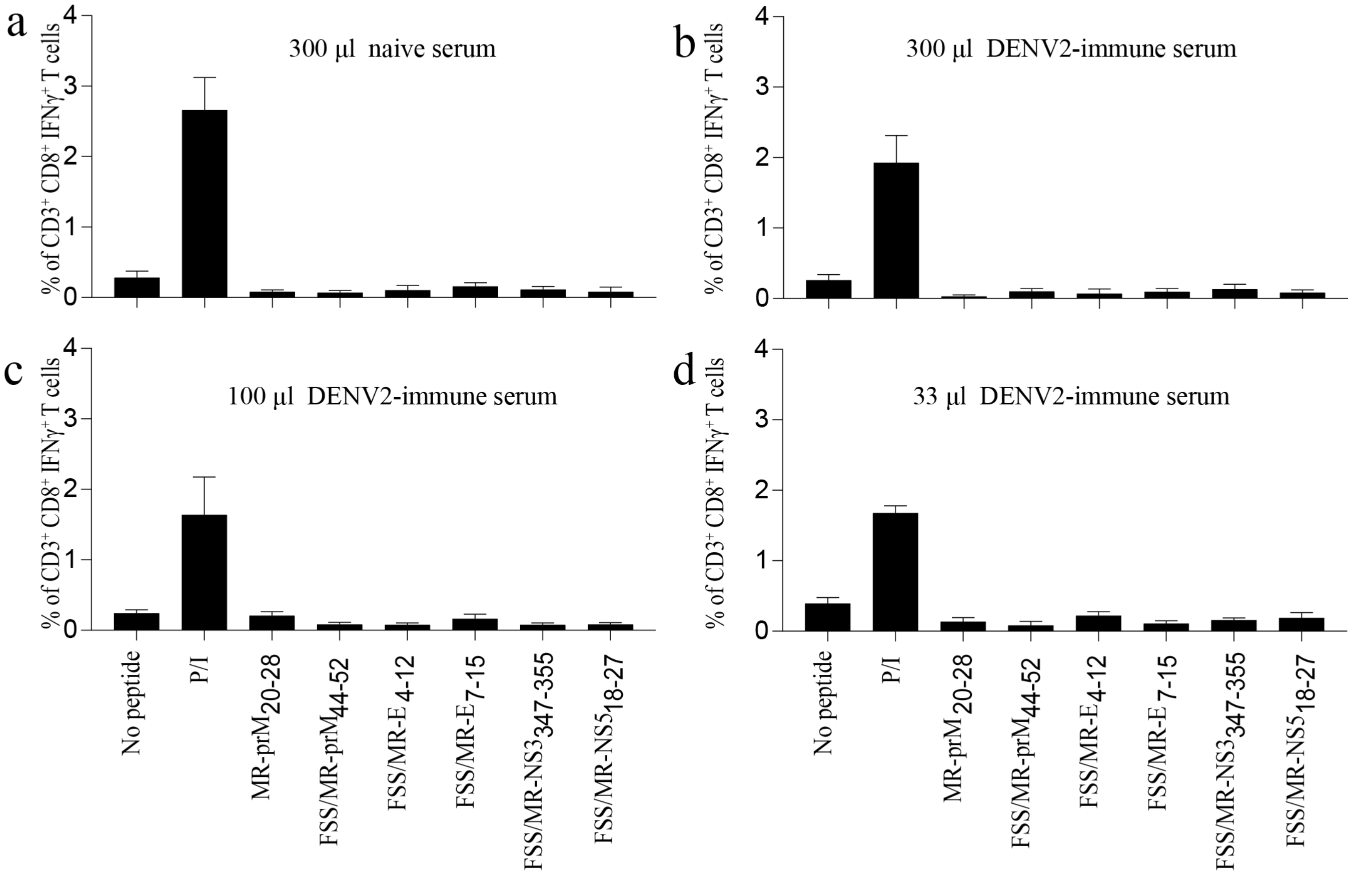

Supplementary Figure 4 Lack of DENV2-immune CD8$^+$ T cell response in *Ifnar1$^{-/-}$* mice receiving DENV2-immune sera

Different volumes of naïve (**a**) or DENV2-immune *Ifnar1$^{-/-}$* mouse sera (**b-d**) were passively transferred (retro-orbital route) to naïve recipient *Ifnar1$^{-/-}$* mice (n=5). Three days post ZIKV challenge (1×10^4 FFU of ZIKV FSS13025, retro-orbital route) peptide-specific CD8$^+$ T cell response was detected by ICS assay. Data are from one single experiment and are expressed as mean ± SEM. A Kruskal-Wallis one-way ANOVA was performed, and all *P* values were >0.05. Supplementary Tables 1 and 2 provide exact values of *n* and *P*.

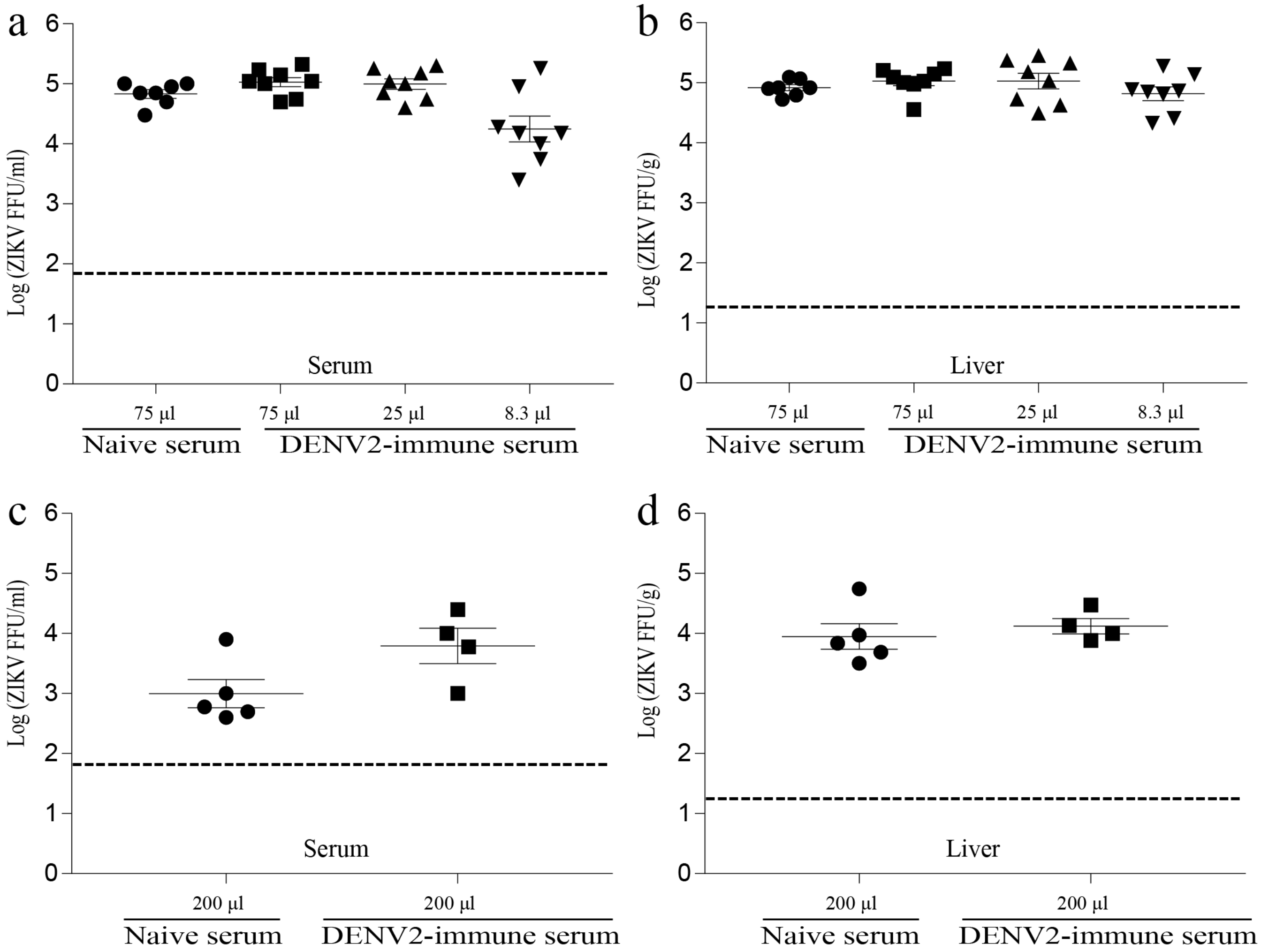

Supplementary Figure 5 Lack of ZIKV control by DENV2-immune sera in WT mice

Different volumes of naïve WT and DENV2-immune WT mouse sera were passively transferred to 2-week-old naïve WT mice (after intraperitoneal injection of 1 mg of IFNAR1-blocking mAb MAR1-5A3) (**a-b**) and 4-week-old naive WT mice (after intraperitoneal injection of 2 mg of IFNAR1-blocking mAb MAR1-5A3) (**c-d**) one day before ZIKV challenge. Three days post ZIKV challenge (5×10^4 FFU of ZIKV FSS13025, intraperitoneal route in **panels a-b**; 1×10^6 FFU of ZIKV FSS13025, retro-orbital route in **panels c-d**), ZIKV titers in sera and organs were measured using FFA.**In panels a-b**, data are pooled from two independent experiments (n = 3-4 mice per group per experiment), while results in **panels c-d** represent one experiment. Data were expressed as mean ± SEM. **Panels a-b**: A Kruskal-Wallis one-way ANOVA. **Panels c-d**: Two-tailed Mann-Whitney test. All *P* values in this figure were >0.05. Supplementary Tables 1 and 2 provide exact values of *n* and *P*.

Supplementary Reference

1.Elong Ngono, A., et al., *Mapping and Role of the CD8+ T Cell Response During Primary Zika Virus Infection in Mice.* Cell Host Microbe, 2017. **21**(1): p. 35-46.

Supplementary Table 1. Values of *n*

Figures	*n* per group (condition)	
Fig. 1a	8 (Naive + isotype Ab)	
Fig. 1b	8 (DENV2-immune + isotype Ab)	
Fig. 1c	9 (Naive + anti-CD8))	
Fig. 1d	8 (DENV2-immune + anti-CD8)	
Fig. 1e	8 (Naive + isotype Ab)	9 (Naive + anti-CD8)
	8 (DENV2-immune + isotype Ab)	8 (DENV2-immune + anti-CD8)
Fig. 1f	8 (Naive + isotype Ab)	9 (Naive + anti-CD8)
	8 (DENV2-immune + isotype Ab)	8 (DENV2-immune + anti-CD8)
Fig. 1g	8 (Naive + isotype Ab)	9 (Naive + anti-CD8)
	8 (DENV2-immune + isotype Ab)	8 (DENV2-immune + anti-CD8)
Fig. 1h	8 (Naive + isotype Ab)	9 (Naive + anti-CD8)
	8 (DENV2-immune + isotype Ab)	8 (DENV2-immune + anti-CD8)
Fig. 1i	5 (Naive + isotype Ab)	6 (Naive + anti-CD8)
	5 (DENV2-immune + isotype Ab)	5 (DENV2-immune + anti-CD8)
Fig. 2a	10 (Naïve serum + DENV2)	10 (Naïve serum + ZIKV)
	10 (DENV2-immune serum + DENV2)	10 (DENV2-immune serum + ZIKV)
Fig. 2b	8 (300 µl Naive serum)	8 (300 µl DENV2-immune serum)
	8 (100 µl DENV2-immune serum)	8 (33 µl DENV2-immune serum)
Fig. 2c	4 (600 µl Naive serum)	4 (600 µl DENV2-immune serum)
Fig. 3a	8 (1×10^7 naive $CD8^+$ T cells)	8 (1×10^7 DENV2-exposed $CD8^+$ T cells)
Fig. 3b	8 (1×10^7 naive $CD8^+$ T cells)(serum, liver, brain, eye)	8 (1×10^7 DENV2-exposed $CD8^+$ T cells)(serum, liver, brain, eye)
	5 (1×10^7 naive $CD8^+$ T cells)(testis)	5 (1×10^7 DENV2-exposed $CD8^+$ T cells)(testis)
Fig. 4a	11 (1×10^7 naive $CD8^+$ T cells)	9 (1×10^7 DENV2-exposed $CD8^+$ T cells)
Fig. 4b	11 (1×10^7 naive $CD8^+$ T cells)(serum, liver)	9 (1×10^7 DENV2-exposed $CD8^+$ T cells)(serum,liver)
Fig. 5a	10	
Fig. 5b	10	
Fig. 5c	10 (1×10^7 naive $CD8^+$ T cells)	10 (1×10^7 DENV2-exposed $CD8^+$ T cells)
Fig. 5d	10 (1×10^7 naive $CD8^+$ T cells)	10 (1×10^7 DENV2-exposed $CD8^+$ T cells)
Supplementary Fig. 1a	6	
Supplementary Fig. 1b	6	

Supplementary Fig. 1c	6	
Supplementary Fig. 2a	7	
Supplementary Fig. 2b	7	
Supplementary Fig. 2c	7	
Supplementary Fig. 3a	5 (Naive serum + DENV2)	5 (Naive serum + ZIKV)
	5 (DENV2-immune serum + DENV2)	5 (DENV2-immune serum + ZIKV)
Supplementary Fig. 3b	5 (DENV2)	5 (ZIKV)
Supplementary Fig. 4a	5 (300 µl Naive serum)	
Supplementary Fig. 4b	5 (300 µl DENV2-immune serum)	
Supplementary Fig. 4c	5 (100 µl DENV2-immune serum)	
Supplementary Fig. 4d	5 (33 µl DENV2-immune serum)	
Supplementary Fig. 5a	7 (75 µl Naive serum)	8 (75 µl DENV2-immune serum)
	8 (25 µl DENV2-immune serum)	8 (8.3 µl DENV2-immune serum)
Supplementary Fig. 5b	7 (75 µl Naive serum)	8 (75 µl DENV2-immune serum)
	8 (25 µl DENV2-immune serum)	8 (8.3 µl DENV2-immune serum)
Supplementary Fig. 5c	5 (200 µl Naive serum)	4 (200 µl DENV2-immune serum)
Supplementary Fig. 5d	5 (200 µl Naive serum)	4 (200 µl DENV2-immune serum)

Supplementary Table 2. Values of *P*

Figures	p-values (comparison, test)
Fig. 1a	0.001 (P/I vs No peptide, A Kruskal-Wallis one-way ANOVA)
	>0.9999 (MR-prM$_{20-28}$ vs No peptide, A Kruskal-Wallis one-way ANOVA)
	>0.9999 (FSS/MR-prM$_{44-52}$ vs No peptide, A Kruskal-Wallis one-way ANOVA)
	>0.9999 (FSS/MR-E$_{4-12}$ vs No peptide, A Kruskal-Wallis one-way ANOVA)
	>0.9999 (FSS/MR-E$_{7-15}$ vs No peptide, A Kruskal-Wallis one-way ANOVA)
	>0.9999 (FSS/MR-NS3$_{347-355}$ vs No peptide, A Kruskal-Wallis one-way ANOVA)
	>0.9999 (FSS/MR-NS5$_{18-27}$ vs No peptide, A Kruskal-Wallis one-way ANOVA)
Fig. 1b	<0.0001 (P/I vs No peptide, A Kruskal-Wallis one-way ANOVA)
	>0.9999 (MR-prM$_{20-28}$ vs No peptide, A Kruskal-Wallis one-way ANOVA)
	0.002 (FSS/MR-prM$_{44-52}$ vs No peptide, A Kruskal-Wallis one-way ANOVA)
	0.0195 (FSS/MR-E$_{4-12}$ vs No peptide, A Kruskal-Wallis one-way ANOVA)
	0.0028 (FSS/MR-E$_{7-15}$ vs No peptide, A Kruskal-Wallis one-way ANOVA)
	0.0017 (FSS/MR-NS3$_{347-355}$ vs No peptide, A Kruskal-Wallis one-way ANOVA)
	0.1035 (FSS/MR-NS5$_{18-27}$ vs No peptide, A Kruskal-Wallis one-way ANOVA)
Fig. 1c	0.0142 (P/I vs No peptide, A Kruskal-Wallis one-way ANOVA)
	>0.9999 (MR-prM$_{20-28}$ vs No peptide, A Kruskal-Wallis one-way ANOVA)
	>0.9999 (FSS/MR-prM$_{44-52}$ vs No peptide, A Kruskal-Wallis one-way ANOVA)
	>0.9999 (FSS/MR-E$_{4-12}$ vs No peptide, A Kruskal-Wallis one-way ANOVA)
	>0.9999 (FSS/MR-E$_{7-15}$ vs No peptide, A Kruskal-Wallis one-way ANOVA)
	>0.9999 (FSS/MR-NS3$_{347-355}$ vs No peptide, A Kruskal-Wallis one-way ANOVA)
	>0.9999 (FSS/MR-NS5$_{18-27}$ vs No peptide, A Kruskal-Wallis one-way ANOVA)
Fig. 1d	<0.0001 (P/I vs No peptide, A Kruskal-Wallis one-way ANOVA)
	0.55 (MR-prM$_{20-28}$ vs No peptide, A Kruskal-Wallis one-way ANOVA)
	0.0048 (FSS/MR-prM$_{44-52}$ vs No peptide, A Kruskal-Wallis one-way ANOVA)
	0.0035 (FSS/MR-E$_{4-12}$ vs No peptide, A Kruskal-Wallis one-way ANOVA)
	0.0067 (FSS/MR-E$_{7-15}$ vs No peptide, A Kruskal-Wallis one-way ANOVA)
	0.0069 (FSS/MR-NS3$_{347-355}$ vs No peptide, A Kruskal-Wallis one-way ANOVA)
	0.3898 (FSS/MR-NS5$_{18-27}$ vs No peptide, A Kruskal-Wallis one-way ANOVA)
Fig. 1e	0.0003 [(DENV2-immune+isotype) vs (Naive+isotype), two-tailed Mann-Whitney test]
	0.0003 [(DENV2-immune+anti-CD8) vs (Naive+isotype), two-tailed Mann-Whitney test]

	0.0017 [(DENV2-immune+anti-CD8) vs (Naive+anti-CD8), two-tailed Mann-Whitney test]
	0.0002 [(DENV2-immune+anti-CD8) vs (DENV2-immune+isotype), two-tailed Mann-Whitney test]
Fig. 1f	0.0002 [(DENV2-immune+isotype) vs (Naive+isotype), two-tailed Mann-Whitney test]
	0.0002 [(DENV2-immune+anti-CD8) vs (DENV2-immune+isotype), two-tailed Mann-Whitney test]
Fig. 1g	0.0002 [(DENV2-immune+isotype) vs (Naive+isotype), two-tailed Mann-Whitney test]
	0.0002 [(DENV2-immune+anti-CD8) vs (DENV2-immune+isotype), two-tailed Mann-Whitney test]
Fig. 1h	0.0006 [(DENV2-immune+isotype) vs (Naive+isotype), two-tailed Mann-Whitney test]
	0.0003 [(DENV2-immune+anti-CD8) vs (DENV2-immune+isotype), two-tailed Mann-Whitney test]
Fig. 1i	0.0079 [(DENV2-immune+isotype) vs (Naive+isotype), two-tailed Mann-Whitney test]
	0.0079 [(DENV2-immune+anti-CD8) vs (DENV2-immune+isotype), two-tailed Mann-Whitney test]
Fig. 2b	0.0051 [(300 µl Naive serum) vs (300 µl DENV2-immune serum), A Kruskal-Wallis one-way ANOVA] (serum)
	0.4041 [(300 µl Naive serum) vs (100 µl DENV2-immune serum), A Kruskal-Wallis one-way ANOVA] (serum)
	0.1080 [(300 µl Naive serum) vs (33 µl DENV2-immune serum), A Kruskal-Wallis one-way ANOVA] (serum)
	0.1285 [(300 µl Naive serum) vs (300 µl DENV2-immune serum), A Kruskal-Wallis one-way ANOVA] (liver)
	0.6913 [(300 µl Naive serum) vs (100 µl DENV2-immune serum), A Kruskal-Wallis one-way ANOVA] (liver)
	0.5223 [(300 µl Naive serum) vs (33 µl DENV2-immune serum), A Kruskal-Wallis one-way ANOVA] (liver)
	>0.9999 [(300 µl Naive serum) vs (300 µl DENV2-immune serum), A Kruskal-Wallis one-way ANOVA] (brain)
	>0.9999 [(300 µl Naive serum) vs (100 µl DENV2-immune serum), A Kruskal-Wallis one-way ANOVA] (brain)
	>0.9999 [(300 µl Naive serum) vs (33 µl DENV2-immune serum), A Kruskal-Wallis one-way ANOVA] (brain)
	>0.9999 [(300 µl Naive serum) vs (300 µl DENV2-immune serum), A Kruskal-Wallis one-way ANOVA] (eye)
	>0.9999 [(300 µl Naive serum) vs (100 µl DENV2-immune serum), A Kruskal-Wallis one-way ANOVA] (eye)
	>0.9999 [(300 µl Naive serum) vs (33 µl DENV2-immune serum), A Kruskal-Wallis one-way ANOVA] (eye)
Fig. 2c	0.3429 [(600 µl Naive serum) vs (600 µl DENV2-immune serum), two-tailed Mann-Whitney test] (serum)
	0.4857 [(600 µl Naive serum) vs (600 µl DENV2-immune serum), two-tailed Mann-Whitney test] (liver)
	0.8857 [(600 µl Naive serum) vs (600 µl DENV2-immune serum), two-tailed Mann-Whitney test] (brain)
	0.8857 [(600 µl Naive serum) vs (600 µl DENV2-immune serum), two-tailed Mann-Whitney test] (eye)
Fig. 3a	<0.0001 (P/I vs No peptide, A Kruskal-Wallis one-way ANOVA) (1×10^7 naive $CD8^+$ T cells)
	>0.9999 (MR-prM$_{20-28}$ vs No peptide, A Kruskal-Wallis one-way ANOVA) (1×10^7 naive $CD8^+$ T cells)
	>0.9999 (FSS/MR-prM$_{44-52}$ vs No peptide, A Kruskal-Wallis one-way ANOVA) (1×10^7 naive $CD8^+$ T cells)
	0.099 (FSS/MR-E$_{4-12}$ vs No peptide, A Kruskal-Wallis one-way ANOVA) (1×10^7 naive $CD8^+$ T cells)
	>0.9999 (FSS/MR-E$_{7-15}$ vs No peptide, A Kruskal-Wallis one-way ANOVA) (1×10^7 naive $CD8^+$ T cells)
	0.0953 (FSS/MR-NS3$_{347-355}$ vs No peptide, A Kruskal-Wallis one-way ANOVA) (1×10^7 naive $CD8^+$ T cells)
	0.5635 (FSS/MR-NS5$_{18-27}$ vs No peptide, A Kruskal-Wallis one-way ANOVA) (1×10^7 naive $CD8^+$ T cells)

	0.0169 (P/I vs No peptide, A Kruskal-Wallis one-way ANOVA) (1×10^7 DENV2-exposed CD8$^+$ T cells)
	0.6082 (MR-prM$_{20-28}$ vs No peptide, A Kruskal-Wallis one-way ANOVA) (1×10^7 DENV2-exposed CD8$^+$ T cells)
	0.0002 (FSS/MR-prM$_{44-52}$ vs No peptide, A Kruskal-Wallis one-way ANOVA) (1×10^7 DENV2-exposed CD8$^+$ T cells)
	0.0003 (FSS/MR-E$_{4-12}$ vs No peptide, A Kruskal-Wallis one-way ANOVA) (1×10^7 DENV2-exposed CD8$^+$ T cells)
	0.0135 (FSS/MR-E$_{7-15}$ vs No peptide, A Kruskal-Wallis one-way ANOVA) (1×10^7 DENV2-exposed CD8$^+$ T cells)
	0.0003 (FSS/MR-NS3$_{347-355}$ vs No peptide, A Kruskal-Wallis one-way ANOVA) (1×10^7 DENV2-exposed CD8$^+$ T cells)
	0.0045 (FSS/MR-NS5$_{18-27}$ vs No peptide, A Kruskal-Wallis one-way ANOVA) (1×10^7 DENV2-exposed CD8$^+$ T cells)
Fig. 3b	0.0457 [(1×10^7 DENV2-exposed CD8$^+$ T cells) vs (1×10^7 naive CD8$^+$ T cells), Two-tailed Mann-Whitney test] (serum)
	0.0104 [(1×10^7 DENV2-exposed CD8$^+$ T cells) vs (1×10^7 naive CD8$^+$ T cells), Two-tailed Mann-Whitney test] (liver)
	0.0079 [(1×10^7 DENV2-exposed CD8$^+$ T cells) vs (1×10^7 naive CD8$^+$ T cells), Two-tailed Mann-Whitney test] (testis)
	0.0002 [(1×10^7 DENV2-exposed CD8$^+$ T cells) vs (1×10^7 naive CD8$^+$ T cells), Two-tailed Mann-Whitney test] (brain)
	0.0281 [(1×10^7 DENV2-exposed CD8$^+$ T cells) vs (1×10^7 naive CD8$^+$ T cells), Two-tailed Mann-Whitney test] (eye)
Fig. 4a	0.0001 (P/I vs No peptide, A Kruskal-Wallis one-way ANOVA) (1×10^7 naive CD8$^+$ T cells)
	>0.9999 (MR-prM$_{20-28}$ vs No peptide, A Kruskal-Wallis one-way ANOVA) (1×10^7 naive CD8$^+$ T cells)
	>0.9999 (FSS/MR-prM$_{44-52}$ vs No peptide, A Kruskal-Wallis one-way ANOVA) (1×10^7 naive CD8$^+$ T cells)
	0.9127 (FSS/MR-E$_{4-12}$ vs No peptide, A Kruskal-Wallis one-way ANOVA) (1×10^7 naive CD8$^+$ T cells)
	>0.9999 (FSS/MR-E$_{7-15}$ vs No peptide, A Kruskal-Wallis one-way ANOVA) (1×10^7 naive CD8$^+$ T cells)
	>0.9999 (FSS/MR-NS3$_{347-355}$ vs No peptide, A Kruskal-Wallis one-way ANOVA) (1×10^7 naive CD8$^+$ T cells)
	>0.9999 (FSS/MR-NS5$_{18-27}$ vs No peptide, A Kruskal-Wallis one-way ANOVA) (1×10^7 naive CD8$^+$ T cells)
	<0.0001 (P/I vs No peptide, A Kruskal-Wallis one-way ANOVA) (1×10^7 DENV2-exposed CD8$^+$ T cells)
	0.0896 (MR-prM$_{20-28}$ vs No peptide, A Kruskal-Wallis one-way ANOVA) (1×10^7 DENV2-exposed CD8$^+$ T cells)
	0.0018 (FSS/MR-prM$_{44-52}$ vs No peptide, A Kruskal-Wallis one-way ANOVA) (1×10^7 DENV2-exposed CD8$^+$ T cells)
	<0.0001 (FSS/MR-E$_{4-12}$ vs No peptide, A Kruskal-Wallis one-way ANOVA) (1×10^7 DENV2-exposed CD8$^+$ T cells)
	0.0075 (FSS/MR-E$_{7-15}$ vs No peptide, A Kruskal-Wallis one-way ANOVA) (1×10^7 DENV2-exposed CD8$^+$ T cells)
	0.0021 (FSS/MR-NS3$_{347-355}$ vs No peptide, A Kruskal-Wallis one-way ANOVA) (1×10^7 DENV2-exposed CD8$^+$ T cells)
	0.1116 (FSS/MR-NS5$_{18-27}$ vs No peptide, A Kruskal-Wallis one-way ANOVA) (1×10^7 DENV2-exposed CD8$^+$ T cells)
Fig. 4b	0.0373 [(1×10^7 DENV2-exposed CD8$^+$ T cells) vs (1×10^7 naive CD8$^+$ T cells), Two-tailed Mann-Whitney test] (serum)
	0.0031 [(1×10^7 DENV2-exposed CD8$^+$ T cells) vs (1×10^7 naive CD8$^+$ T cells), Two-tailed Mann-Whitney test] (liver)
Fig. 5c	0.0139 [(1×10^7 DENV2-exposed CD8$^+$ T cells) vs (1×10^7 naive CD8$^+$ T cells), Two-way ANOVA] (day 5)
	<0.0001 [(1×10^7 DENV2-exposed CD8$^+$ T cells) vs (1×10^7 naive CD8$^+$ T cells), Two-way ANOVA] (day 6)
	<0.0001 [(1×10^7 DENV2-exposed CD8$^+$ T cells) vs (1×10^7 naive CD8$^+$ T cells), Two-way ANOVA] (day 7)
Fig. 5d	0.0005 [(1×10^7 DENV2-exposed CD8$^+$ T cells) vs (1×10^7 naive CD8$^+$ T cells), Log rank test)
Supplementary Fig. 1a	0.0007 (P/I vs no peptide, A Kruskal-Wallis one-way ANOVA)

0.0659 (FSS/MR-prM$_{20-28}$ vs no peptide, A Kruskal-Wallis one-way ANOVA)

>0.9999 (prM$_{25-33}$ vs no peptide, A Kruskal-Wallis one-way ANOVA)

0.0075 (FSS/MR-prM$_{44-52}$ vs no peptide, A Kruskal-Wallis one-way ANOVA)

>0.9999 (FSS/MR-prM$_{46-54}$ vs no peptide, A Kruskal-Wallis one-way ANOVA)

0.0312 (FSS/MR-E$_{4-12}$ vs no peptide, A Kruskal-Wallis one-way ANOVA)

0.0212 (FSS/MR-E$_{7-15}$ vs no peptide, A Kruskal-Wallis one-way ANOVA)

>0.9999 (FSS/MR-E$_{48-56}$ vs no peptide, A Kruskal-Wallis one-way ANOVA)

>0.9999 (FSS/MR-E$_{57-65}$ vs no peptide, A Kruskal-Wallis one-way ANOVA)

>0.9999 (FSS-E$_{310-317}$ vs no peptide, A Kruskal-Wallis one-way ANOVA)

>0.9999 (MR-E$_{310-317}$ vs no peptide, A Kruskal-Wallis one-way ANOVA)

>0.9999 (FSS/MR-E$_{345-355}$ vs no peptide, A Kruskal-Wallis one-way ANOVA)

>0.9999 (FSS/MR-E$_{420-428}$ vs no peptide, A Kruskal-Wallis one-way ANOVA)

>0.9999 (MR-E$_{494-502}$ vs no peptide, A Kruskal-Wallis one-way ANOVA)

>0.9999 (FSS/MR-NS1$_{174-183}$ vs no peptide, A Kruskal-Wallis one-way ANOVA)

>0.9999 (FSS/MR-NS2A$_{80-87}$ vs no peptide, A Kruskal-Wallis one-way ANOVA)

>0.9999 (FSS/MR-NS2A$_{179-188}$ vs no peptide, A Kruskal-Wallis one-way ANOVA)

>0.9999 (FSS/MR-NS2B$_{114-122}$ vs no peptide, A Kruskal-Wallis one-way ANOVA)

>0.9999 (FSS/MR-NS3$_{137-145}$ vs no peptide, A Kruskal-Wallis one-way ANOVA)

>0.9999 (FSS/MR-NS3$_{276-284}$ vs no peptide, A Kruskal-Wallis one-way ANOVA)

0.0216 (FSS/MR-NS3$_{347-355}$ vs no peptide, A Kruskal-Wallis one-way ANOVA)

>0.9999 (FSS/MR-NS4A$_{135-142}$ vs no peptide, A Kruskal-Wallis one-way ANOVA)

>0.9999 (FSS/MR-NS4B$_{102-110}$ vs no peptide, A Kruskal-Wallis one-way ANOVA)

0.0165 (FSS/MR-NS5$_{18-27}$ vs no peptide, A Kruskal-Wallis one-way ANOVA)

>0.9999 (FSS-NS5$_{68-77}$ vs no peptide, A Kruskal-Wallis one-way ANOVA)

>0.9999 (MR-NS5$_{68-77}$ vs no peptide, A Kruskal-Wallis one-way ANOVA)

>0.9999 (MR-NS5$_{121-131}$ vs no peptide, A Kruskal-Wallis one-way ANOVA)

>0.9999 (FSS/MR-NS5$_{128-135}$ vs no peptide, A Kruskal-Wallis one-way ANOVA)

>0.9999 (FSS/MR-NS5$_{222-229}$ vs no peptide, A Kruskal-Wallis one-way ANOVA)

>0.9999 (MR-NS5$_{449-557}$ vs no peptide, A Kruskal-Wallis one-way ANOVA)

Supplementary Fig. 1b 0.0007 (P/I vs no peptide, A Kruskal-Wallis one-way ANOVA)

0.0112 (MR-prM$_{20-28}$ vs no peptide, A Kruskal-Wallis one-way ANOVA)

>0.9999 (prM$_{25-33}$ vs no peptide, A Kruskal-Wallis one-way ANOVA)

0.0095 (FSS/MR-prM$_{44-52}$ vs no peptide, A Kruskal-Wallis one-way ANOVA)

	>0.9999 (FSS/MR-prM$_{46-54}$ vs no peptide, A Kruskal-Wallis one-way ANOVA)
	0.1293 (FSS/MR-E$_{4-12}$ vs no peptide, A Kruskal-Wallis one-way ANOVA)
	0.0221 (FSS/MR-E$_{7-15}$ vs no peptide, A Kruskal-Wallis one-way ANOVA)
	>0.9999 (FSS/MR-E$_{48-56}$ vs no peptide, A Kruskal-Wallis one-way ANOVA)
	>0.9999 (FSS/MR-E$_{57-65}$ vs no peptide, A Kruskal-Wallis one-way ANOVA)
	>0.9999 (FSS-E$_{310-317}$ vs no peptide, A Kruskal-Wallis one-way ANOVA)
	>0.9999 (MR-E$_{310-317}$ vs no peptide, A Kruskal-Wallis one-way ANOVA)
	>0.9999 (FSS/MR-E$_{345-355}$ vs no peptide, A Kruskal-Wallis one-way ANOVA)
	>0.9999 (FSS/MR-E$_{420-428}$ vs no peptide, A Kruskal-Wallis one-way ANOVA)
	>0.9999 (MR-E$_{494-502}$ vs no peptide, A Kruskal-Wallis one-way ANOVA)
	>0.9999 (FSS/MR-NS1$_{174-183}$ vs no peptide, A Kruskal-Wallis one-way ANOVA)
	>0.9999 (FSS/MR-NS2A$_{80-87}$ vs no peptide, A Kruskal-Wallis one-way ANOVA)
	>0.9999 (FSS/MR-NS2A$_{179-188}$ vs no peptide, A Kruskal-Wallis one-way ANOVA)
	>0.9999 (FSS/MR-NS2B$_{114-122}$ vs no peptide, A Kruskal-Wallis one-way ANOVA)
	>0.9999 (FSS/MR-NS3$_{137-145}$ vs no peptide, A Kruskal-Wallis one-way ANOVA)
	>0.9999 (FSS/MR-NS3$_{276-284}$ vs no peptide, A Kruskal-Wallis one-way ANOVA)
	0.0158 (FSS/MR-NS3$_{347-355}$ vs no peptide, A Kruskal-Wallis one-way ANOVA)
	>0.9999 (FSS/MR-NS4A$_{135-142}$ vs no peptide, A Kruskal-Wallis one-way ANOVA)
	>0.9999 (FSS/MR-NS4B$_{102-110}$ vs no peptide, A Kruskal-Wallis one-way ANOVA)
	0.011 (FSS/MR-NS5$_{18-27}$ vs no peptide, A Kruskal-Wallis one-way ANOVA)
	>0.9999 (FSS-NS5$_{68-77}$ vs no peptide, A Kruskal-Wallis one-way ANOVA)
	>0.9999 (MR-NS5$_{68-77}$ vs no peptide, A Kruskal-Wallis one-way ANOVA)
	>0.9999 (MR-NS5$_{121-131}$ vs no peptide, A Kruskal-Wallis one-way ANOVA)
	>0.9999 (FSS/MR-NS5$_{128-135}$ vs no peptide, A Kruskal-Wallis one-way ANOVA)
	>0.9999 (FSS/MR-NS5$_{222-229}$ vs no peptide, A Kruskal-Wallis one-way ANOVA)
	>0.9999 (MR-NS5$_{449-557}$ vs no peptide, A Kruskal-Wallis one-way ANOVA)
Supplementary Fig. 1c	0.0003 (P/I vs no peptide, A Kruskal-Wallis one-way ANOVA)
	0.0369 (MR-prM$_{20-28}$ vs no peptide, A Kruskal-Wallis one-way ANOVA)
	>0.9999 (prM$_{25-33}$ vs no peptide, A Kruskal-Wallis one-way ANOVA)
	0.0087 (FSS/MR-prM$_{44-52}$ vs no peptide, A Kruskal-Wallis one-way ANOVA)
	>0.9999 (FSS/MR-prM$_{46-54}$ vs no peptide, A Kruskal-Wallis one-way ANOVA)
	0.015 (FSS/MR-E$_{4-12}$ vs no peptide, A Kruskal-Wallis one-way ANOVA)
	0.0172 (FSS/MR-E$_{7-15}$ vs no peptide, A Kruskal-Wallis one-way ANOVA)

	>0.9999 (FSS/MR-E_{48-56} vs no peptide, A Kruskal-Wallis one-way ANOVA)
	>0.9999 (FSS/MR-E_{57-65} vs no peptide, A Kruskal-Wallis one-way ANOVA)
	>0.9999 (FSS-$E_{310-317}$ vs no peptide, A Kruskal-Wallis one-way ANOVA)
	>0.9999 (MR-$E_{310-317}$ vs no peptide, A Kruskal-Wallis one-way ANOVA)
	>0.9999 (FSS/MR-$E_{345-355}$ vs no peptide, A Kruskal-Wallis one-way ANOVA)
	>0.9999 (FSS/MR-$E_{420-428}$ vs no peptide, A Kruskal-Wallis one-way ANOVA)
	>0.9999 (MR-$E_{494-502}$ vs no peptide, A Kruskal-Wallis one-way ANOVA)
	>0.9999 (FSS/MR-$NS1_{174-183}$ vs no peptide, A Kruskal-Wallis one-way ANOVA)
	>0.9999 (FSS/MR-$NS2A_{80-87}$ vs no peptide, A Kruskal-Wallis one-way ANOVA)
	>0.9999 (FSS/MR-$NS2A_{179-188}$ vs no peptide, A Kruskal-Wallis one-way ANOVA)
	>0.9999 (FSS/MR-$NS2B_{114-122}$ vs no peptide, A Kruskal-Wallis one-way ANOVA)
	>0.9999 (FSS/MR-$NS3_{137-145}$ vs no peptide, A Kruskal-Wallis one-way ANOVA)
	>0.9999 (FSS/MR-$NS3_{276-284}$ vs no peptide, A Kruskal-Wallis one-way ANOVA)
	0.0255 (FSS/MR-$NS3_{347-355}$ vs no peptide, A Kruskal-Wallis one-way ANOVA)
	>0.9999 (FSS/MR-$NS4A_{135-142}$ vs no peptide, A Kruskal-Wallis one-way ANOVA)
	>0.9999 (FSS/MR-$NS4B_{102-110}$ vs no peptide, A Kruskal-Wallis one-way ANOVA)
	0.0172 (FSS/MR-$NS5_{18-27}$ vs no peptide, A Kruskal-Wallis one-way ANOVA)
	>0.9999 (FSS-$NS5_{68-77}$ vs no peptide, A Kruskal-Wallis one-way ANOVA)
	>0.9999 (MR-$NS5_{68-77}$ vs no peptide, A Kruskal-Wallis one-way ANOVA)
	>0.9999 (MR-$NS5_{121-131}$ vs no peptide, A Kruskal-Wallis one-way ANOVA)
	>0.9999 (FSS/MR-$NS5_{128-135}$ vs no peptide, A Kruskal-Wallis one-way ANOVA)
	>0.9999 (FSS/MR-$NS5_{222-229}$ vs no peptide, A Kruskal-Wallis one-way ANOVA)
	>0.9999 (MR-$NS5_{449-557}$ vs no peptide, A Kruskal-Wallis one-way ANOVA)
Supplementary Fig 2a	<0.0001 (P/I vs no peptide, A Kruskal-Wallis one-way ANOVA)
	0.31 (DENV2-prM_{44-52} vs no peptide, A Kruskal-Wallis one-way ANOVA)
	<0.0001 (DENV2-E_{4-12} vs no peptide, A Kruskal-Wallis one-way ANOVA)
	0.0217 (DENV2-E_{7-15} vs no peptide, A Kruskal-Wallis one-way ANOVA)
	0.046 (DENV2-$NS3_{347-355}$ vs no peptide, A Kruskal-Wallis one-way ANOVA)
	>0.9999 (DENV2-$NS5_{18-27}$ vs no peptide, A Kruskal-Wallis one-way ANOVA)
Supplementary Fig 2b	<0.0001 (P/I vs no peptide, A Kruskal-Wallis one-way ANOVA)
	0.0088 (DENV2-prM_{44-52} vs no peptide, A Kruskal-Wallis one-way ANOVA)
	0.0005 (DENV2-E_{4-12} vs no peptide, A Kruskal-Wallis one-way ANOVA)
	>0.9999 (DENV2-E_{7-15} vs no peptide, A Kruskal-Wallis one-way ANOVA)

	<0.9999 (DENV2-NS3$_{347-355}$ vs no peptide, A Kruskal-Wallis one-way ANOVA)
	>0.9999 (DENV2-NS5$_{18-27}$ vs no peptide, A Kruskal-Wallis one-way ANOVA)
Supplementary Fig 2c	<0.0001 (P/I vs no peptide, A Kruskal-Wallis one-way ANOVA)
	0.0367 (DENV2-prM$_{44-52}$ vs no peptide, A Kruskal-Wallis one-way ANOVA)
	<0.0001 (DENV2-E$_{4-12}$ vs no peptide, A Kruskal-Wallis one-way ANOVA)
	0.0656 (DENV2-E$_{7-15}$ vs no peptide, A Kruskal-Wallis one-way ANOVA)
	0.1023 (DENV2-NS3$_{347-355}$ vs no peptide, A Kruskal-Wallis one-way ANOVA)
	>0.9999 (DENV2-NS5$_{18-27}$ vs no peptide, A Kruskal-Wallis one-way ANOVA)
Supplementary Fig 3b	0.0079 (DENV2 vs ZIKA, two-tailed Mann-Whitney test)
Supplementary Fig 4a	0.7063 (P/I vs No peptide, A Kruskal-Wallis one-way ANOVA)
	>0.9999 (MR-prM$_{20-28}$ vs No peptide, A Kruskal-Wallis one-way ANOVA)
	0.9534 (FSS/MR-prM$_{44-52}$ vs No peptide, A Kruskal-Wallis one-way ANOVA)
	>0.9999 (FSS/MR-E$_{4-12}$ vs No peptide, A Kruskal-Wallis one-way ANOVA)
	>0.9999 (FSS/MR-E$_{7-15}$ vs No peptide, A Kruskal-Wallis one-way ANOVA)
	>0.9999 (FSS/MR-NS3$_{347-355}$ vs No peptide, A Kruskal-Wallis one-way ANOVA)
	0.5447 (FSS/MR-NS5$_{18-27}$ vs No peptide, A Kruskal-Wallis one-way ANOVA)
Supplementary Fig 4b	0.8364 (P/I vs No peptide, A Kruskal-Wallis one-way ANOVA)
	0.2968 (MR-prM$_{20-28}$ vs No peptide, A Kruskal-Wallis one-way ANOVA)
	>0.9999 (FSS/MR-prM$_{44-52}$ vs No peptide, A Kruskal-Wallis one-way ANOVA)
	0.3855 (FSS/MR-E$_{4-12}$ vs No peptide, A Kruskal-Wallis one-way ANOVA)
	>0.9999 (FSS/MR-E$_{7-15}$ vs No peptide, A Kruskal-Wallis one-way ANOVA)
	>0.9999 (FSS/MR-NS3$_{347-355}$ vs No peptide, A Kruskal-Wallis one-way ANOVA)
	>0.9999 (FSS/MR-NS5$_{18-27}$ vs No peptide, A Kruskal-Wallis one-way ANOVA)
Supplementary Fig 4c	>0.9999 (P/I vs No peptide, A Kruskal-Wallis one-way ANOVA)
	>0.9999 (MR-prM$_{20-28}$ vs No peptide, A Kruskal-Wallis one-way ANOVA)
	0.3366 (FSS/MR-prM$_{44-52}$ vs No peptide, A Kruskal-Wallis one-way ANOVA)
	0.2270 (FSS/MR-E$_{4-12}$ vs No peptide, A Kruskal-Wallis one-way ANOVA)
	>0.9999 (FSS/MR-E$_{7-15}$ vs No peptide, A Kruskal-Wallis one-way ANOVA)
	0.2194 (FSS/MR-NS3$_{347-355}$ vs No peptide, A Kruskal-Wallis one-way ANOVA)
	0.3057 (FSS/MR-NS5$_{18-27}$ vs No peptide, A Kruskal-Wallis one-way ANOVA)
Supplementary Fig 4d	>0.9999 (P/I vs No peptide, A Kruskal-Wallis one-way ANOVA)
	0.4173 (MR-prM$_{20-28}$ vs No peptide, A Kruskal-Wallis one-way ANOVA)
	0.073 (FSS/MR-prM$_{44-52}$ vs No peptide, A Kruskal-Wallis one-way ANOVA)

	>0.9999 (FSS/MR-E_{4-12} vs No peptide, A Kruskal-Wallis one-way ANOVA)
	0.2498 (FSS/MR-E_{7-15} vs No peptide, A Kruskal-Wallis one-way ANOVA)
	>0.9999 (FSS/MR-$NS3_{347-355}$ vs No peptide, A Kruskal-Wallis one-way ANOVA)
	>0.9999 (FSS/MR-$NS5_{18-27}$ vs No peptide, A Kruskal-Wallis one-way ANOVA)
Supplementary Fig 5a	0.4246 [(75 µl Naive serum) vs (75 µl DENV2-immune serum), A Kruskal-Wallis one-way ANOVA] (serum)
	0.6555 [(75 µl Naive serum) vs (25 µl DENV2-immune serum), A Kruskal-Wallis one-way ANOVA] (serum)
	0.5957 [(75 µl Naive serum) vs (8.3 µl DENV2-immune serum), A Kruskal-Wallis one-way ANOVA] (serum)
Supplementary Fig 5b	0.7553 [(75 µl Naive serum) vs (75 µl DENV2-immune serum), A Kruskal-Wallis one-way ANOVA] (liver)
	0.9329 [(75 µl Naive serum) vs (25 µl DENV2-immune serum), A Kruskal-Wallis one-way ANOVA] (liver)
	>0.9999 [(75 µl Naive serum) vs (8.3 µl DENV2-immune serum), A Kruskal-Wallis one-way ANOVA] (liver)
Supplementary Fig 5c	0.0714 [(200 µl Naive serum) vs (200 µl DENV2-immune serum), two-tailed Mann-Whitney test] (serum)
Supplementary Fig 5d	0.2857 [(200 µl Naive serum) vs (200 µl DENV2-immune serum), two-tailed Mann-Whitney test] (liver)

Supplementary Table 3. H-2^{b}-restricted CD8^{+} T cell epitopes in ZIKV[a]

Peptides[b]	Sequences
MR-prM$_{20-28}$	ISFATTLGV
prM$_{25-33}$	TLGMNKCYI
FSS/MR-prM$_{44-52}$	ATMSYECPM
FSS/MR-prM$_{46-54}$	MSYECPML
FSS/MR-E$_{4-12}$	IGVSNRDFV
FSS/MR-E$_{7-15}$	SNRDFVEGM
FSS/MR-E$_{48-56}$	TTSVNMAEV
FSS/MR-E$_{57-65}$	RSYCYEASI
FSS-E$_{310-317}$	AAFTFTKV
MR-E$_{310-317}$	AAFTFTKI
FSS/MR-E$_{345-355}$	MAVDMQTLTPV
FSS/MR-E$_{420-428}$	RMAVLGDTA
MR-E$_{494-502}$	VMIFLSTAV
FSS/MR-NS1$_{174-183}$	YSLECDPAVI
FSS/MR-NS2A$_{80-87}$	VSFIFRAN
FSS/MR-NS2A$_{179-188}$	SVKKNLPFVM
FSS/MR-NS2B$_{114-122}$	AAGAWYVYV
FSS/MR-NS3$_{137-145}$	VVIKNGSYV
FSS/MR-NS3$_{276-284}$	SSIAARGYI
FSS/MR-NS3$_{347-355}$	PSVRNGNEI
FSS/MR-NS4A$_{135-142}$	IMVAVGLL
FSS/MR-NS4B$_{102-110}$	SQLTPLTLI
FSS/MR-NS5$_{18-27}$	CAEAPNMKVI
FSS-NS5$_{68-77}$	SSLVNGVVRL
MR-NS5$_{68-77}$	SSLINGVVRL
MR-NS5$_{121-131}$	RQVMNIVSSWL
FSS/MR-NS5$_{128-135}$	SSWLWKEL

FSS/MR-NS5$_{222-229}$	RAIWYMWL
MR-NS5$_{449-557}$	TGWSNWEEV

[a]Peptides were published on Cell Host Microbe (Elong Ngono, A., et al. 2017, 21(1):35-46).

[b]MR and FSS denote MR766 and FSS13025, respectively.
